# Acclimation of thermal tolerance in juvenile plants from three biomes is suppressed when extremes co-occur

**DOI:** 10.1093/conphys/coae027

**Published:** 2024-05-22

**Authors:** Rosalie J Harris, Philippa R Alvarez, Callum Bryant, Verónica F Briceño, Alicia M Cook, Andrea Leigh, Adrienne B Nicotra

**Affiliations:** Research School of Biology, The Australian National University, 134 Linnaeus Way, Acton ACT 2601, Canberra, Australian Capital Territory, Australia; School of Life Sciences, University of Technology Sydney, PO Box 123, Broadway, Sydney NSW 2007, Australia; Research School of Biology, The Australian National University, 134 Linnaeus Way, Acton ACT 2601, Canberra, Australian Capital Territory, Australia; Research School of Biology, The Australian National University, 134 Linnaeus Way, Acton ACT 2601, Canberra, Australian Capital Territory, Australia; School of Life Sciences, University of Technology Sydney, PO Box 123, Broadway, Sydney NSW 2007, Australia; School of Life Sciences, University of Technology Sydney, PO Box 123, Broadway, Sydney NSW 2007, Australia; Research School of Biology, The Australian National University, 134 Linnaeus Way, Acton ACT 2601, Canberra, Australian Capital Territory, Australia

**Keywords:** Chlorophyll fluorescence, cold tolerance, common conditions, heat tolerance, plant communities, PSII

## Abstract

Given the rising frequency of thermal extremes (heatwaves and cold snaps) due to climate change, comprehending how a plant’s origin affects its thermal tolerance breadth (TTB) becomes vital. We studied juvenile plants from three biomes: temperate coastal rainforest, desert and alpine. In controlled settings, plants underwent hot days and cold nights in a factorial design to examine thermal tolerance acclimation. We assessed thermal thresholds (*T*_crit-hot_ and *T*_crit-cold_) and TTB. We hypothesized that (i) desert species would show the highest heat tolerance, alpine species the greatest cold tolerance and temperate species intermediate tolerance; (ii) all species would increase heat tolerance after hot days and cold tolerance after cold nights; (iii) combined exposure would broaden TTB more than individual conditions, especially in desert and alpine species. We found that biome responses were minor compared to the responses to the extreme temperature treatments. All plants increased thermal tolerance in response to hot 40°C days (*T*_crit-hot_ increased by ~3.5°C), but there was minimal change in *T*_crit-cold_ in response to the cold −2°C nights. In contrast, when exposed to both hot days and cold nights, on average, plants exhibited an antagonistic response in TTB, where cold tolerance decreased and heat tolerance was reduced, and so we did not see the bi-directional expansion we hypothesized. There was, however, considerable variation among species in these responses. As climate change intensifies, plant communities, especially in transitional seasons, will regularly face such temperature swings. Our results shed light on potential plant responses under these extremes, emphasizing the need for deeper species-specific thermal acclimation insights, ultimately guiding conservation efforts.

## Teaser

In a changing climate, our study on plants from diverse biomes revealed unexpected responses to combined hot days and cold nights, reducing thermal tolerance breadth. While biome differences were minimal, overall responses to treatments varied significantly, highlighting the need for conservation efforts, and detailed species-specific thermal acclimation studies.

## Introduction

Extreme weather and temperature anomalies can constitute important selective events and instigate regulation and acclimation responses of individuals as well as adaptation of species (Anderson and Song, 2020; Li *et al.,* 2021). Increasing intensities and frequencies of extreme heat and cold events associated with global climate change are challenging many plant species’ thermal tolerance thresholds (Trisos *et al.,* 2020; Geange *et al.,* 2021). The physiological damage caused by extreme weather events can differentially affect plant species, potentially altering species distributions, resulting in range shifts and changes in community composition (Knight and Ackerly, 2002; Curtis *et al.,* 2016; O’Sullivan *et al.,* 2017; Zhu *et al.,* 2018; Geange *et al.,* 2021; Posch *et al.,* 2022). Most experiments investigating plant thermal tolerance have focused on responses to high- or low-temperature extremes alone (Zhu *et al.,* 2018; Leon-Garcia and Lasso, 2019; Geange *et al.,* 2021; Saini *et al.,* 2022). However, climate change is affecting the frequency and duration of temperature extremes, which may extend growing seasons, reduce snow cover and increase frost exposure (Menzel *et al.,* 2020; Slatyer *et al.,* 2022). Therefore, there is a need to consider both high- and low-temperature tolerances in concert. Characterizing plant species thermal tolerance breadth (TTB)—the thermal range between high- and low-temperature tolerances—and the acclimation potential thereof may be important indicators of differential resilience and vulnerability in plants from different biomes to changing climates (Valladares *et al.,* 2007; Geange *et al.,* 2021).

Implications of climate change for plant performance and persistence could differ across biomes, given distinct patterns of climate variability. Species that live in benign climatic conditions, such as temperate coastal rainforests, are expected to have a narrower thermal tolerance range because of the relatively stable year-round thermal conditions (Sunday *et al.,* 2011; Molina-Montenegro and Naya, 2012). Likewise, plants in these benign climates may be less likely to be exposed to major variations in temperature over the coming decades. In contrast, impacts might be particularly severe in more extreme habitats. For example, plants in alpine regions can not only experience extreme cold but also reach relatively high leaf temperatures (Salisbury and Spomer, 1964; Buchner *et al.,* 2015). Declines in snow cover are extending the length of the growing season (Zhang *et al.,* 2019; Jabis *et al.,* 2020), while the loss of the thermal buffer that snow provides threatens greater exposure to extreme cold for sensitive alpine plant communities (Zhang, 2005; Larcher *et al.,* 2010; Semenchuk *et al.,* 2013). These changes have already been linked to altered flowering patterns, diminished biomass, range shifts and the decline of frost-sensitive species (Ball *et al.,* 1991; Ball *et al.,* 1997; Briceño *et al.,* 2014; Zohner *et al.,* 2020). At the other extreme, plants living in arid regions, which already exist in highly variable conditions, are experiencing changes in precipitation in concert with hot days and, at times, very cold nights (Díaz *et al.,* 2019). When plants are water limited, they will often conserve water by closing their stomata, and this comes with the significant risk of an increase in leaf temperature, inducing cellular and tissue damage (Aparecido *et al.,* 2020; Cook *et al.,* 2021; Marchin *et al.,* 2022). The evolutionary strategies of these plants to endure such extremes offer insights into potential adaptive responses to future climatic conditions.

The likelihood of co-occurring extreme heat and cold events may seem low, but species growing in highly seasonal climates are exposed to wide temperature ranges, particularly in spring and autumn. These seasons can cause significant stress to plants as they have not yet acclimated to hot (in spring) or cold (in autumn) conditions. For example, early frosts can be especially dangerous for alpine plants in spring, where leaves at midday can reach 38°C, then experience a − 2°C frost the following morning (Briceño *et al.,* 2014). Spring can also be a stressful time for arid species as they transition out of a winter-acclimated state where unseasonably hot days and heatwaves can cause damage to multiple organs of the plant (Allstadt *et al.,* 2015). Plants exhibit varied responses, contingent on the intensity and duration of thermal stress and the growth conditions, and genetic factors inherent to the individual (Wang *et al.,* 2016; Ruehr *et al.,* 2019). Over evolutionary periods, adaptation through trait selection for plasticity has ensured intergenerational survival (Nicotra *et al.,* 2015). However, given the swift pace of climate change and extreme conditions, evolutionary adaptation might lag, making plasticity through acclimation paramount (Way and Yamori, 2014; Nicotra *et al.,* 2015).

While thermal tolerance can be defined in several ways, one commonly used means of assaying thermal tolerance in plants is via temperature-dependent chlorophyll fluorescence, which indicates the (high or low) temperature sensitivities of photosystem II in the photosynthetic apparatus (Schreiber and Berry, 1977). Temperature-dependent fluorescence (*T*–*F*_0_ curves) can generate metrics of the upper (*T*_crit-hot_) and lower (*T*_crit-cold_) critical limits, allowing for comparisons of thermal tolerance across a range of different experiments, species, locations and treatments, shedding light on global patterns of plant responses to extremes, particularly to climate change (Knight and Ackerly, 2002; Curtis *et al.,* 2016; O’Sullivan *et al.,* 2017; Sastry and Barua, 2017; Zhu *et al.,* 2018; Feeley *et al.,* 2020; Arnold *et al.,* 2021; Harris *et al.*, 2022; Posch *et al.,* 2022). Most of the plant thermal tolerance literature focuses on species’ response to freezing or heatwaves (sometimes in association with elevated CO_2_ and/or drought); there is, however a paucity of studies on the combined effects of heat and cold exposure (Geange *et al.,* 2021). Further, field surveys of thermal tolerance do not allow for controlled factorial manipulation of extreme events, often relying on seasonal comparisons over timescales of weeks to months, and the potential for species’ short-term acclimation is rarely captured. Previous work has shown that heat tolerance increases with mean annual growth temperature and latitude (O’Sullivan *et al.,* 2017), suggesting that variation across distinct biomes would result in very different heat tolerance temperatures. Knight and Ackerly (2002) found greater differences in thermal tolerance in field-grown plants from the desert compared to their coastal region congenerics, but these innate differences were mostly diminished when plants were grown under common conditions. Thus, we do not know the extent to which differences in thermal tolerance observed among biomes reflect *in situ* acclimation or innate species differences.

It is yet unclear whether thermal acclimation to heat and cold stress happens in tandem or separately. A bi-directional expansion of thermal tolerance might arise when exposure to one type of stress also bolsters tolerance to the other, as is the case for heat shock protein upregulation (Wang *et al.,* 2003; Swindell *et al.,* 2007). Conversely, heat and cold protective responses may be independent, one catering to heat and the other to cold (Knight and Knight, 2001). If so, we would only expect a wider TTB when both heat stress and cold stress occur concurrently. We used a fully factorial common conditions experiment to investigate short-term acclimation response of the *T*_crit-hot_ and *T*_crit-cold_ and TTB (the difference between these two) to diurnal extremes of heat and cold of 24 species from three thermally distinct biomes: temperate coastal rainforest, alpine and desert. We hypothesized that (i) even under common conditions, species from the desert biome would have the highest heat tolerances, and alpine species would be the most cold tolerant, with the temperate species having moderate thermal tolerance; (ii) plants would increase their heat tolerance after exposure to hot days and shift their cold tolerance to colder (more negative) temperatures after exposure to cold nights; (iii) that (a) the combination of both stressors (hot days with cold nights) would increase thermal tolerance more so than either alone, such that TTB would be greatest in plants exposed to both, and (b) the plants from the more variable environments (alpine and desert biomes) would be more capable of bi-directional expansion of TTB than temperate species.

## Materials and Methods

### Species selection

Species from temperate rainforest, alpine and desert biomes were selected to compare biome responses to hot days and cold nights using temperature-dependent increases in chlorophyll *a* fluorescence. For each biome, we selected eight species for which seeds were available in conservation seed banks based on the following criteria: accessions stored for <20 years, accessions collected within a 50-km radius within areas of three distinct Australian biomes: alpine (Kosciuszko National Park, NSW), desert (Bourke, NSW) or temperate coastal rainforest (Wollongong, NSW). When more than one accession was available, we used the most recently collected seed for each species. For all species, we used seed sourced from a single accession. We also sought to include representatives of key families within each biome and of families common to all biomes (Supplementary Table S1).

For most species, 25 seeds were sown onto each of two Petri dishes containing 0.8% water-agar. Seed was obtained from the Australian National Botanic Gardens Seed Bank and the Australian Botanic Gardens Australian PlantBank. Several alpine species were cold stratified at 4°C for 6 weeks to alleviate dormancy before transfer to germination incubators (Thermoline Scientific, Melbourne, NSW Australia), which simulated the optimum germination conditions for the species. Seed from some species required scarification, smoke treatment or gibberellic acid before placement in incubators (Supplementary Table S2 for details on germination strategies). As soon as seeds germinated, they were transferred to the Australian National University and potted in 4 × 4-cm pots with native mix and 3 cm of seed-raising mix at the surface to help delicate roots establish. Seedlings were grown under common conditions in glasshouses exposed to natural circadian rhythm at 25°C day/15°C night cycles for 3–5 months depending on germination time, and were watered daily. Seedlings were fertilized every 2 weeks with 10-ml Seasol low phosphorus (for Australian native plants) liquid fertilizer.

Some species had low germination rates, and so seven of the 24 species were purchased from the Monaro Native Tree Nursery NSW and two species were purchased from the Bodalla Nursery NSW (Supplementary Table S1). All nursery-raised plants were grown from seed collected within the focal biomes and were approximately 3 months old at time of purchase. Nursery-raised plants were acclimated along with plants grown from seed for two months under common conditions prior to commencement of the experiment.

### Experimental design

Experiments were conducted in Conviron plant growth chambers (Model PCG20; Conviron Asia Pacific PTY Ltd, Grovedale, Victoria) at the Plant Phenomics Facility, Commonwealth Scientific Industrial Research Organisation (CSIRO), Canberra, from 5 to 19 April 2021 (35°16′21.6″S 149°06′57.3″E). We used a fully factorial experimental design with 3 biomes × 8 species per biome × 5 replicates (one per block) × 4 temperature treatments = 480 plants. The five experimental blocks were separated temporally by 1–2 days to stagger the fluorescence assays. Plants of similar height and diameter were blocked together with the tallest plants in Block 1 and the shortest plants in Block 5 to minimize the overtopping of smaller plants by larger ones. Because we were interested in the effects of extreme temperatures, we designed the treatments to reflect spring extremes, that young plants (e.g. early in their second growing season) would be exposed to the more extreme environments. Daytime temperature regimes were based on the average of three consecutive days above the maximum temperature of early growth season conditions for a time relative to each biome. Night temperatures were based on the average spring minimum, for Australian alpine and desert regions. Plants of each block were randomly allocated to temperature regimes for 5 days; thermal tolerance was measured on Days 3 and 5: the benign reference treatment (control) was maintained at temperatures in which the plants were raised (25°C days/15°C nights), the hot days treatment subjected plants to hot days and benign nights (40°C days/15°C nights), the cold nights treatment subjected plants to benign days and cold nights (25°C days/−2°C nights), the combination treatment challenged plants with both hot days and cold nights (40°C days/−2°C nights) and all treatments had half-hourly incremental changes to reach target temperatures (Supplementary Fig. S1). We conducted a preliminary trial using a subset of the same species, not those included in the main experiment to determine acclimation was occurring without causing seedling mortality under these thermal regimes; we used electrolyte leakage and *F*_V_/*F*_M_ as indicators of health before proceeding with the experiment to ensure plants would survive the thermal regimes.

To assess whether leaves were reaching the same temperatures as the programmed chamber temperatures, leaf temperature was measured on one individual of most species using type T thermocouples (Omega Engineering) connected to HOBO dataloggers (HOBO UX120; Onset). Leaf temperatures were largely in accord with air temperature, and thus we deemed any modest deviations were not likely to undermine the efficacy of the treatments (Supplementary Fig. S2). Air temperature and relative humidity were also measured by sensors within the chambers. Light levels in the chambers were programmed to 0 μmol between 7:30 pm and 6:30 am, ramping by 100 μmol h^−1^ to 800 μmol at 10:30 am, then maintained at this point until ramping down from 4:30 pm for a total of 12 h of daylight. To prevent freezing damage to the roots in the −2°C treatments, we insulated the roots using an emergency foil blanket wrapped around the base of each tray. For each block, all four treatments occurred simultaneously, one treatment per chamber, and each block received the same period of treatment exposure. Plants were kept well watered throughout.

### Thermal tolerance assays

Initial *F*_V_/*F*_M_ was measured for baseline status of maximum quantum yield prior to treatment implementation to determine the health of photosynthetic tissue of a subset of individuals before entry to chambers for Blocks 1, 3 and 5 using a PEA meter (Hansatech Instruments, Ltd). Leaves were dark adapted for 30 min before *F*_V_/*F*_M_ measurements were measured at 9:00 every morning. There were no significant declines in *F*_V_/*F*_M_ in any of the treatments throughout the experiment, indicating that plants remained healthy with minimal damage throughout (Supplementary Fig. S3).

Assays of thermal tolerance were measured between 10:00 am and noon, when temperatures were between 15°C and 21°C in all chambers on Days 3 and 5 of the experiment. Leaf discs of 1 cm^2^ were punched from one leaf per plant and placed into pill boxes moistened with florist foam to maintain turgor until thermal tolerance assays. We set up two Maxi Pulse Amplitude Modulating (PAM) systems (Heinz Walz GmbH, Effeltrich, Germany), one for *T*_crit-hot_ and one for *T*_crit-cold_ measurements. Each PAM was placed directly above a Peltier plate (CP-121HT; TE-Technology, Inc., Michigan, USA; 152 × 152-mm surface), regulated by a temperature ramp controller (TC-36-25; TE-Technology, Inc.) and powered by a fixed-voltage power supply (PS-24-13; TE-Technology, Inc.). Cooling rates were programmed to 15°C h^−1^ from 20°C to −25°C and basal fluorescence (*F*_0_) measured every 20 s. Heating rates were programmed to 30°C h^−1^ from 20°C to 65°C; see Arnold *et al.* (2021) for specifications of PAM setup and parameterizations. Leaf discs were placed on a paper array with unique grid references made up of 48 cells, and location of leaf samples within the grid was randomized for each run. A type T thermocouple (Omega Engineering) was attached to the abaxial side of each leaf and monitored with a 48-channel dataTaker DT85 (Lontek, Australia), logging every 5 s. The critical temperatures during heating and cooling, *T*_crit-hot_ and *T*_crit-cold_, were defined as the breakpoint between the slow and fast-rise phases of basal fluorescence (Arnold *et al.,* 2021).

### Statistical analysis

Values of *T*_crit_ were extracted using the segmented package in R (code available at https://github.com/pieterarnold/Tcrit-extraction). TTB was calculated as the difference between *T*_crit-hot_ and *T*_crit-cold_. Linear mixed-effects models (lmer packages in R) were used for all analyses, with *T*_crit-hot_, *T*_crit-cold_ or TTB as the response variables. Biome, hot days and cold nights and their interactions were included as fixed effects. Block was a random effect, with a combined variable of measurement day (Day 3 or 5) nested within species. We adopted this nesting structure because repeatability tests on Days 3 and 5 for each treatment yielded a high *R*^2^ and slopes between 0.9 and 1, suggesting highly repeatable measurements. Therefore, rather than including measurement day as a random effect (only two levels), we nested the day within species, which accounts for the measurements days not being independent. All analyses were performed in R version 4.0.2 (R Core Team, 2018).

## Results

### Biome effects were small, but in some cases significant

**Figure 1:**
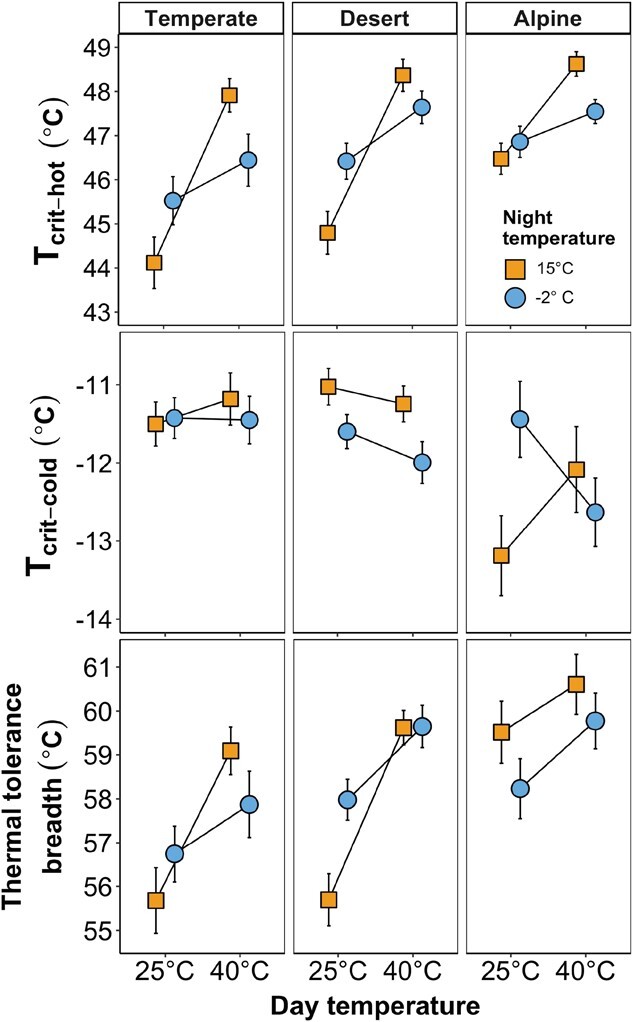
Interaction plots for *T*_crit-hot,_*T*_crit-cold_ and TTB across biomes and treatments. (a) Interactions between biome, hot days and cold nights demonstrating that each treatment increased *T*_crit-hot_, with the strongest effect from the hot days treatment while that, overall, the responses across biomes followed the same pattern. (b) Interaction plots for *T*_crit-cold_ indicate that desert and temperate plants showed little change after treatments, whereas alpine plants followed a different pattern and decreased cold tolerance after exposure to any combination of hot days or cold nights. (c) Interaction plots for TTB show hot days increased TTB for all biomes but to a much lesser extent for the alpine group, in which TTB narrowed in response to exposure to cold nights. No plants showed capacity for bi-directional expansion of TTB. Values are linear mixed effects model means ± SE.

Among the plants under benign thermal regimes, alpine plants had a surprisingly high *T*_crit-hot_ of 46.9 ± 0.35°C (Supplementary Table S3), which was 2.3°C higher than the temperate reference (Fig. 1a, Supplementary Table S3). These alpine plants also had the greatest cold tolerance of −13.2 ± 0.51°C, with a *T*_crit-cold_ 1.7°C more negative than the temperate reference (Fig. 1b, Supplementary Table S3). This meant that overall, amongst plants grown under benign conditions, temperate and desert plants had a narrower inherent breadth at 55.7 ± 0.75°C and 55.7 ± 0.60°C, respectively, while alpine plants had the widest TTB of 59.5 ± 0.70°C (Fig. 1c, Table S3). Nonetheless, these differences, while statistically significant, amount to less than a 10% difference in TTB among biomes.

### Acclimation response influenced more by temperature stress than biome

*T*_crit-hot_ increased in plants exposed to the hot days treatment. This was most pronounced in the temperate and desert biomes with an increase of 3.8 ± 0.3°C and 3.6 ± 0.3°C, respectively (see Supplementary Table S3 for biome and treatment means). The alpine plants showed the least potential to acclimate in response to hot days with an increase of only 2.2 ± 0.2°C (but also had a high baseline *T*_crit-hot_). In response to cold nights, plants from the temperate and desert biomes both increased *T*_crit-hot_, while alpine plants remained unchanged. After exposure to the combination of hot days/cold nights, all plants, regardless of biome, increased *T*_crit-hot_ at a reduced capacity of 1–2°C less compared to hot days alone (Fig. 1a, Table 1, Supplementary Table S3).

**Table 1:** Summary table of linear mixed effects models to test for changes in *T*_crit-hot_, *T*_crit-cold_ and TTB for treatments and biomes. Bold indicates significance at *P* < 0.05; *, *P* <0.05; **, *P* < 0.01; ***, *P* < 0.001.

	***T***_**crit-hot**_		***T***_**crit-cold**_		**TTB**	
**Predictors**	***F***	***P***	***F***	***P***	***F***	***P***
Biome	1.200	0.301	1.079	0.339	1.3402	0.261
Hot day	**119.098**	**<0.001** ^***^	0.165	0.684	**73.707**	**<0.001** ^***^
Cold night	0. 0.192	0.661	0.153	0.695	0.191	0.662
Biome × hot day	2.7267	0.065	0.690	0.501	1.979	0.138
Biome × cold night	1.1905	0.303	**4.782**	**0.008** ^**^	**5.571**	**0.003** ^**^
Hot day × cold night	**29.846**	**<0.001** ^***^	**7.569**	**0.005** ^**^	**5.942**	**0.014** ^*^
Biome × hot day × cold night	0.677	0.508	**4.178**	**0.015** ^*^	1.930	0.145

Bold indicates significance at *P* < 0.05, *P* < 0.01 and *P* < 0.001 from lmerTest package in R.

As for *T*_crit-cold_, following the cold nights, the alpine plants had either no change or a decrease (less negative) in *T*_crit-cold_, becoming less cold tolerant. Similarly, after exposure to the combination of hot days/cold nights, alpine plants became less cold tolerant compared to the other biomes, yielding a significant three-way interaction between biomes × hot days × cold nights (Fig. 1b, Table 1). Desert and temperate plants did not change.

TTB: After the hot days treatment, plants in all biomes increased their TTB, but only by 1.1 ± 0.6°C for alpine plants compared to 3.4 ± 0.5°C for temperate and 4 ± 0.3°C for desert plants (Fig. 1c, Table 1, Supplementary Table S3). Interestingly, among the alpine plants, the shift in *T*_crit-cold_ meant that TTB narrowed after exposure to cold nights, whereas the TTB for temperate and desert biomes did not change (Fig. 1c, Table 1, Supplementary Table S3). All biomes exhibited much greater shifts in TTB after exposure to hot days as compared to cold nights alone as indicated by significant two-way interactions between hot days and cold nights (Fig. 1c, Table 1). We asked whether the combination treatment might broaden TTB and found that the combination of the hot days with cold nights led to an overall narrower TTB compared to hot days alone, but improved TTB compared to cold nights alone (Supplementary Fig. S4). Our results indicate that biome was not a statistically significant predictor of bi-directional expansion of TTB in response to thermal stress.

**Figure 2:**
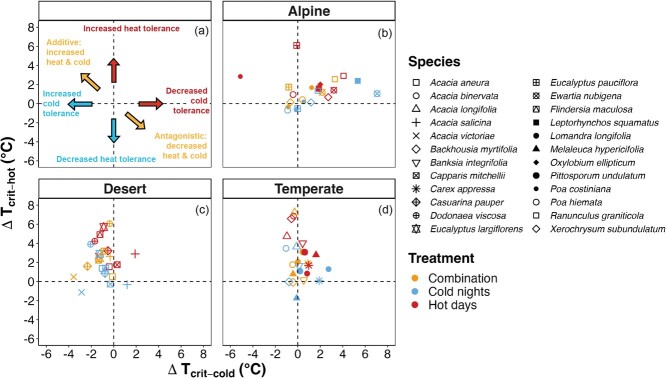
The hypothesized (a) vs observed (b–d) difference between *T*_crit-hot_ and *T*_crit-cold_ for each treatment for species in each biome relative to their respective reference treatment. Based on the resource cost of increased tolerance, our expectation was that shifts in tolerance would represent different trade-offs, depending on treatment. We had hypothesized that hot days would shift species up towards higher heat tolerance on the *y*-axis with no effect on the *x*-axis (panel a, upper two quadrants), while we expected cold nights to improve cold tolerance on the *x*-axis with no effect on the *y*-axis position (left-hand quadrants). We did not expect any treatment to lead to reduction in both heat and cold tolerance (lower right quadrant). Finally, we expected the combination treatment would be additive, where species responses would reside in the upper left-hand quadrant. We found that alpine species (b) tended to become increasingly heat tolerant at the cost of cold tolerance, regardless of treatment. The desert species (c) showed the additive effect that we expected for the combination treatment, but for all treatments. The temperate species (d) became increasingly heat tolerant but did not deviate much in their cold tolerance.

We also noted that the species differences (a random effect) explained a large portion of the variation in all our models. We therefore also visually examined (without statistical inference) what that variation looked like and assessed whether there was a difference in the distribution of cases in which a species–treatment combination showed increases in either or both *T*_crit-hot_ and *T*_crit-cold_ in response to the temperature treatments (Fig. 2 and see Supplementary Table S4 for individual species means). We plotted the difference in heat and cold tolerances for each treatment relative to the reference treatment for each species as a qualitative assessment of the species-level responses. Our visual assessment of the shifts shows that there was some indication of trade-offs in thermal tolerance in the alpine species; i.e. an increase in heat tolerance was associated with a decrease in cold tolerance. In contrast, in the desert species, heat or cold or the combination of both treatments all generally corresponded with increases in both heat and cold tolerance. In the temperate species, there was some increase in heat tolerance in response to warming but little change in cold tolerance (Fig. 2).

## Discussion

We explored the acclimation of thermal tolerance limits in juvenile plants from three contrasting biomes—temperate coastal rainforest, alpine and desert. We predicted that desert plants would have the highest heat tolerance, but instead we found broad thermal tolerance in all biomes, and alpine plants were surprisingly the most heat tolerant on average, while temperate and desert plants exhibited somewhat more modest *T*_crit-hot_. As expected, alpine plants had the greatest inherent cold tolerance in terms of *T*_crit-cold_ and thus greatest TTB, lending partial support to our hypothesis that plants of extreme environments would have greater TTB. We found that acclimation to hot days via upwards shifts in heat tolerance was consistent across all biomes, in line with our second hypothesis. However, exposure to cold nights did not improve the plants’ cold tolerance, deviating from our expectations. Lastly, we predicted that the combined exposure to hot days and cold nights would incur a bi-directional expansion of TTB, but we found highly variable effects. On average, there was no change or even reduced thermal tolerance, largely led by a reduction in cold tolerance, leading us to reject our third hypothesis. Our findings suggest that on average, plant species may have a limited ability to acclimate to their full extent if they are exposed to sudden co-occurring hot days and cold nights; this could be particularly important for desert and alpine plants during spring and autumn, when temperature ranges are widest and plants have not yet fully acclimated to those conditions. Below we first explore biome effects (or lack thereof) before considering acclimation to altered temperature regimes and its implication in a climate change and conservation and management context.

### Biome of origin had limited influence on the TTB or magnitude of acclimation to thermal extremes

The TTBs exhibited here are notable in that they greatly exceeded the thermal range of the species’ environment of origin, even for plants grown under benign and relatively invariant conditions. Although we did discern minor biome-specific differences in response to our experimental conditions (Fig. 2), the overarching impact was the temperature treatments themselves, indicating a strong, convergent acclimatory effect across these diverse biomes. This acclimatory effect may explain apparent biome differences in baseline tolerance from field data. For example, the high heat tolerance of alpine plants under common conditions may be the result of higher growth temperatures in the glasshouse relative to the mean for alpine plants, causing their *T*_crit-hot_ to be higher before they were even exposed to an extreme event. By contrast, the moderate tolerance of desert and temperate species, whose mean heat and cold tolerance under benign condition were very similar to one another, could reflect acclimation to benign growth temperatures relative to their biome of origin. Thus, by comparing these three quite distinct biomes, our results suggest that the local thermal conditions and/or ontogenetic changes may explain more of the variation in thermal tolerance than ecological history of conditions at seed origin.

Multiple ecological, physiological and evolutionary factors interplay to determine plant distribution. Microclimates, biotic interactions, life stage sensitivities and evolutionary histories each play a role in defining these niches (Comita and Engelbrecht, 2009; Scherrer and Körner, 2010; Savolainen *et al.,* 2013). The diversity within biome classifications significantly influences plant adaptations and responses to environmental challenges. For instance, the environmental dynamics of temperate tropical forests, with their unique temperature fluctuations and seasonality, differ from non-tropical temperate forests (Choury *et al.,* 2022). Similarly, deserts like the Sonoran and the Mojave or Great Basin exhibit distinct environmental conditions that shape their flora (Beatley, 1974; Medeiros and Drezner, 2012). These variations extend beyond mere geographic differences, impacting evolutionary trajectories, particularly in aspects like cellular membrane composition and thermal stability (Sultan, 2000; Lambers and Oliveira, 2008). Recognizing these differences is crucial for understanding plant responses to temperature extremes. Our study sheds light on biome-specific responses but also underscores the importance of understanding how different environments within the same biome category can lead to distinct evolutionary adaptations in plants, especially under the stress of climate change. This perspective is vital for future research aimed at comprehensively understanding plant adaptations and resilience in diverse global ecosystems.

Juvenile plants, although possessing broad TTBs, might have specific requirements or sensitivities that adult plants do not, marking a potential bottleneck during establishment. Additionally, physiological trade-offs and distributional lags might further narrow the realized niche of the species compared to their fundamental niche potential as inferred by TTB alone (Franks *et al.,* 2014; Perez *et al.,* 2023). Time of seedlings emergence varies across these biomes, and so perhaps alpine plants are readily acclimated to cope with cold conditions early in establishment, while temperate ones are not. However, that all these juvenile plants were able to rapidly respond and acclimate to these short-term stressors is a good indicator of plasticity within photosystem II (PSII). Future research should holistically examine these complex factors, not just at the leaf level but to the whole plant and across broader ontogenetic stages, to decipher the observed patterns in plant distribution relative to their thermal tolerances.

### Strong acclimation response to extreme heat vs cold

All our plants increased their heat tolerance in response to the treatments relative to the benign ones to some extent—even surprisingly, when exposed to cold nights. We also found interactions between hot days and cold nights for each metric of thermal tolerance, suggesting both hot days and cold nights were significantly contributing to either extent or direction of changes in *T*_crit_. Notably, even as juveniles, which are often assumed to be a highly susceptible life stage, these plants exhibited an impressive ability to cope with both high and low temperatures. The magnitude of change in *T*_crit_ between heat and cold tolerance was stark, with changes of up to 4.5°C after exposure to hot days for heat tolerance, while cold tolerance barely changed for desert and temperate plants, with alpine plants exhibiting a counter-intuitive reduction in cold tolerance (Fig. 2). The change in *T*_crit-hot_ for species of the desert and temperate biomes was large relative to the smaller shift of the alpine plants, which scarcely shifted their baseline *T*_crit-hot_ when exposed to the hot days treatment. Our findings align with Zhu *et al.* (2018) who found no difference in *T*_crit-hot_ between summer and winter acclimated cold origin plants. Indeed, it is interesting that our plants from all biomes had an upper limit of *T*_crit-hot_ at 48°C, regardless of their tolerance at benign conditions. This inherent resilience, even when grown in common conditions without prior exposure to extremes, underscores the ecological significance of the capacity to adapt and is crucial for the future viability of these species.

Adaptations to aridity, such as smaller leaves and thicker cell walls, may inadvertently increase the freezing tolerance of the desert species by enhancing supercooling capacity. (Lintunen *et al.,* 2013; Körner, 2016; Dörken *et al.,* 2020). However, our study focused on woody arid zone plants for logistical reasons. We recognize that grasses and forbs are important components of the arid zone flora and may be less sclerophyllous or have larger leaves than the drought-resistant woody plants considered here. Therefore, our findings on the convergent nature of stress-tolerant traits in temperature and drought-stress tolerance may not fully represent the entire spectrum of desert plant adaptations; future research encompassing a broader scope of growth form and leaf trait variation would provide a more comprehensive understanding of biome-specific stress tolerance mechanisms. We recognize the compounding effects of climate change such as increased uncertainty in precipitation in concert with temperature variability. Prior research has demonstrated the potential for interactive effects, both priming and exacerbating (Ostmeyer *et al.,* 2020; Zhou *et al.,* 2020), and thus investigating the combined effects of thermal stress and drought is crucial for understanding plant adaptive mechanisms under complex climate change scenarios and for guiding conservation and ecosystem management strategies.

Previous studies have predominately looked at the effects of heat on *T*_crit_ of PSII rather than cold, and so we have very few studies for comparison of our cold tolerance findings (Andrew *et al.*, 2022). Cold tolerance appears to be more variable than heat tolerance (Sunday *et al.,* 2011; Araújo *et al.,* 2013), in line with the lack of consistent response to cold and the strong response to heat between upper heat and cold limits that we found here. Large variation in cold tolerance is likely the response of individual species’ ability to adjust metabolic processes according to changes in their thermal environment, and especially in response to seasonal changes (Pagter and Arora, 2013; Fürtauer *et al.,* 2019). Changes in heat tolerance, by contrast, tend to be fast and reasonably more consistent across species and even higher-level taxa. The convergent nature of plants’ upper thermal limits in response to heat may be more directly attributed to the immediate and unequivocal constraints imposed by physical laws, as extreme heat can rapidly lead to organismal death (Sharkey, 2005). This contrasts with the effects of cold temperatures, where the impacts on chemical reactions are less immediate, allowing for a more gradual acclimation process and recovery (Theocharis *et al.,* 2012). Indeed, literature on chilling effects, especially in agricultural species, indicates that cold acclimation can occur at temperatures well above freezing, up to 4°C (Kocsy *et al.,* 2001; Kuk *et al.,* 2003; Ruelland *et al.,* 2009). Given this, we had good reason to expect that the temperatures in our study were sufficiently low to trigger a cold response. However, shortening of the photoperiod can also induce cold hardiness and acclimation; perhaps because we did not shorten the photoperiod, the cold acclimation response could have been somewhat suppressed (Mac Irving and Lanphear, 1967). There is also a growing body of evidence to suggest that plants can perceive cold through changes in plasma membrane fluidity, leading to increases in Ca^2+^ to the cytosol. This is perceived by the plant at temperatures as warm as 4°C and occurs over just a few days to initiate cold acclimation (Knight *et al.,* 1996; Örvar *et al.,* 2000). Regardless, our results were unintuitive, especially given how strongly the plants all responded to the extreme heat conditions. In addition, the alpine plants whose cold tolerance reduced after exposure to cold nights, especially in environments where snow reduction might expose plants to more frequent and severe freezing temperatures, point to the need for further investigation into cold tolerance, time of exposure and severity.

### Exposure to a combination of hot days and cold nights resulted in suppressed acclimation

To our knowledge, there are no studies that have investigated the effects of hot days accompanied by cold nights in a fully factorial design before. We hypothesized that the effects of cold nights and hot days would be additive, such that TTB would be greatest in plants exposed to both extremes, but instead we found that on average, the combined treatment appeared to suppress heat tolerance by ~1.5°C relative to hot days alone. As for cold tolerance, the combined treatment did improve cold tolerance relative to the cold nights treatment for alpine and desert plants (Fig. 1). The combined treatment seemed to be antagonistic in the direction of heat tolerance, where heat tolerance was increased, but not as much as heat alone, and cold tolerance was suppressed (Fig. 2). It is important to clarify that such combinations of hot and cold temperatures within a single day are not unusual. In certain biomes, like the alps and desert, it is indeed common for clear, hot days to be followed by extremely cold nights. In these settings, leaf temperatures in full sun can dramatically exceed air temperatures, making it entirely realistic for a leaf to experience a swing from 35°C in the daytime to −2°C overnight during spring or autumn. The temperate rainforest species studied here, by contrast, are often found in shaded understory conditions or breezy coastal areas, where leaf temperatures are moderated by the microclimate, and therefore, this combination stressor is much less likely, if ever present. We found that on average, plants had suppressed acclimation in response to the combination treatment, but species-specific responses are more complicated than this, with some showing signs of an antagonistic response while others were additive (Fig. 2b–d). Therefore, further investigation into the traits and physiology of species-specific responses is warranted in the future.

To elicit bi-directional widening of TTB relative to the control and even the individual stressors, the treatments would need to trigger pathways that simultaneously upregulate heat and cold tolerance. This could mean triggering two separate pathways or generating additive responses in common pathways. For example, if both hot and cold stresses engage similar heat shock proteins (HSP) signalling pathways, plants could potentially enhance their cold tolerance when exposed to hot conditions (Suzuki *et al.,* 2012). At the cellular level, heat tolerance often depends on factors like sugar concentrations around the chloroplast and membrane integrity (Seemann *et al.,* 1986; Lazár and Ilík, 1997). Osmotic adjustment is another mechanism that could aid both heat and cold tolerance, adding another layer of complexity to our understanding (Munns, 2002). While membrane rigidity, driven by the conversion of violaxanthin to zeaxanthin, enhances heat tolerance (Havaux and Tardy, 1996), cold tolerance seems to benefit from osmotic adjustment as well as membrane fluidity (Santarius, 1992). Our study specifically investigates PSII, a vital component of the photosynthesis pathway highly sensitive to temperature changes. For a comprehensive understanding of plant responses to temperature extremes, future research should also consider traits beyond PSII, including water-use efficiency, stomatal conductance and leaf and root morphology. Comprehensive research into the fitness costs or benefits of thermal acclimation, particularly in relation to PSII and other essential traits, will be vital for understanding these complex responses.

## Conclusions

Although our experimental conditions of a 40°C day and a −2°C night may seem like a rare event in nature, it is not unrealistic or unusual for plants to experience very high leaf temperatures that exceed air temperatures in the day, accompanied by very cold nights. Our study reveals that plants often exhibit a suppressed acclimation response to these co-occurring extremes, contrasting with their response to singular stressors. This suppressed acclimation could lead to increased susceptibility to frost injury or heat stress, diminished reproductive success, increased mortality and competitive disadvantages, potentially causing cascading effects in ecosystems. Such findings underscore the importance of future research addressing thermal tolerance to simultaneous heat and cold extremes, particularly for plants in highly variable climates. Understanding the drivers of thermal acclimation across species and their strategies to cope with varying stresses is crucial for enhancing plant adaptability to climate change. Insights from this research can inform adaptive management in restored landscapes (Tudor *et al.,* 2023) and improve restoration strategies and outcomes in severely disturbed landscapes (Tomlinson *et al.,* 2022; Valliere *et al.,* 2022). By identifying mechanisms of resilience to extreme events like heatwaves and cold snaps, we can better target conservation efforts towards species and ecosystems most at risk.

## Supplementary Material

Web_Material_coae027

## Data Availability

Data are available in the Dryad Digital Repository, https://doi.org/10.5061/dryad.cz8w9gjbg.
