# Supplementary material for: Acclimation of thermal tolerance in juvenile plants from three biomes is suppressed when extremes co-occur
Source: Conserv Physiol. 2024 May 22;12(1):coae027. doi: 10.1093/conphys/coae027 (PMC11756708; doi:10.1093/conphys/coae027)
Supplement: Web_Material_coae027 [file web_material_coae027.pdf]

Supporting information

Supplementary Table 1. Species used in study with a * to denote purchased from nursery

**Biome**	**Species**	**Family**	**Growth Form**
Alpine	*Eucalyptus pauciflora**	Myrtaceae	Tree
Alpine	*Poa costiniana**	Poaceae	Grass
Alpine	*Ranunculus graniticola**	Ranunculaceae	Herb
Alpine	*Oxylobium ellipticum*	Fabaceae	Shrub
Alpine	*Leptorhynchos squamatus**	Asteraceae	Herb
Alpine	*Poa heimata**	Poaceae	Grass
Alpine	*Xerochrysum subundulatum**	Asteraceae	Herb
Alpine	*Ewartia nubigena*	Asteraceae	Herb
Desert	*Flindersia maculosa*	Rutaceae	Tree
Desert	*Capparis mitchelli*	Capparaceae	Tree
Desert	*Eucalyptus largiflorens*	Myrtaceae	Tree
Desert	*Acacia salicina*	Fabaceae	Tree
Desert	*Casuarina pauper*	Casuarinaceae	Tree
Desert	*Acacia victoriae*	Fabaceae	Tree
Desert	*Dodonaea viscosa*	Sapindaceae	Shrub
Desert	*Acacia aneura*	Fabaceae	Tree
Temperate	*Acacia longifolia*	Fabaceae	Shrub/Tree
Temperate	*Lomandra longifolia**	Asparagaceae	Herb
Temperate	*Backhousia myrtifolia*	Myrtaceae	Tree
Temperate	*Banksia integrifolia*	Proteaceae	Tree
Temperate	*Pittosporum undulatum**	Pittosporaceae	Tree
Temperate	*Melaleuca hypericifolia**	Myrtaceae	Shrub
Temperate	*Carex appressa*	Cyperaceae	Sedge
Temperate	*Acacia binervata*	Fabaceae	Shrub/Tree

Supplementary Table 2. Germination strategy for each species that was grown from seed.

Biome	Species	Pre-treat	Gibberellic acid	Temp and light conditions
Desert	*Acacia aneura*	Scarify	No	20°C, 12/12 light
Desert	*Acacia salicina*	Scarify	No	20°C, 12/12 light
Desert	*Capparis mitchellii*	No	No	20°C, 12/12 light
Desert	*Casuarina pauper*	No	No	25°C, 12/12 light
Desert	*Dodonaea viscosa*	Nicking	No	25°C, 12/12 light
Desert	*Eucalyptus largiflorens*	No	No	25°C, 12/12light
Desert	*Acacia victoriae*	Hot water	No	25/15°C, 12/12 light
Desert	*Flindersia maculosa*	Removed wing	No	25°C, 12/12 light
Temperate	*Carex appressa*	No	No	25/15°C, 12/12 light
Temperate	*Banksia integrifolia*	No	No	25°C, 12/12 light
Temperate	*Backhousia myrtifolia*	No	No	25°C, 12/12 light
Temperate	*Acacia binervata*	Scarify	No	25°C, 12/12 light
Temperate	*Acacia longifolia*	Scarify	No	25°C, 12/12 light
Alpine	*Oxylobium ellipticum*	Hot water	No	25/15°C, 12/12 light
Alpine	*Ewartia nubigena*	No	Yes	25/15°C, 12/12 light

Supplementary Table 3. Absolute means and standard errors in °C for each metric of thermal tolerance for all combinations of biomes and treatments.

**Treatment**	**Biome**	$T_{\text{crit-hot}}$		$T_{\text{crit-cold}}$		**TTB**		
		Mean	***SE***	***Mean***	***SE***	***Mean***	***SE***	***n***
**Reference**	Alpine	46.474	0.352	-13.187	0.281	59.516	0.708	78
	Temperate	44.118	0.583	-11.501	0.281	55.678	0.749	80
	Desert	44.797	0.484	-11.025	0.233	55.695	0.595	74
**Hot Days**	Alpine	48.623	0.277	-12.084	0.548	60.606	0.685	76
	Temperate	47.912	0.378	-11.181	0.333	59.094	0.542	80
	Desert	48.366	0.364	-11.245	0.229	59.622	0.391	80
**Cold Nights**	Alpine	46.858	0.352	-11.441	0.485	58.233	0.681	75
	Temperate	45.524	0.544	-11.425	0.260	56.742	0.638	80
	Desert	46.419	0.406	-11.598	0.217	57.979	0.465	80
**Combination**	Alpine	47.545	0.271	-12.631	0.440	59.773	0.634	78
	Temperate	46.443	0.591	-11.450	0.305	57.872	0.756	80
	Desert	47.641	0.369	-11.994	0.267	59.647	0.481	80

Supplementary Table 4. Species means in °C for each metric $T_{\text{crit-hot}}$ and $T_{\text{crit-cold}}$ and TTB by control and biome. Values are means ± SE.

Species	Treatment	Biome	*n*	Mean $T_{\text{crit-hot}}$	SE	Mean $T_{\text{crit-cold}}$	SE	Mean TTB	SE
Acacia binervata	Cold	Temp	10	45.802	1.213	-10.98	0.509	56.673	1.153
Acacia binervata	Combo	Temp	10	45.028	1.268	-10.157	0.675	55.185	0.967
Acacia binervata	Hot	Temp	10	48.848	0.666	-10.307	0.918	59.155	0.854
Acacia binervata	Ref	Temp	10	42.014	1.447	-9.931	0.504	51.896	1.48
Acacia longifolia	Cold	Temp	10	44.212	1.113	-12.078	0.927	56.29	1.361
Acacia longifolia	Combo	Temp	10	47.807	0.585	-12.26	0.849	60.067	1.353
Acacia longifolia	Hot	Temp	10	45.298	0.983	-12.939	0.942	58.237	1.277
Acacia longifolia	Ref	Temp	10	40.57	1.449	-11.972	0.725	52.542	1.385
Backhousia myrtifolia	Cold	Temp	10	39.8	1.795	-10.298	0.425	50.706	1.911
Backhousia myrtifolia	Combo	Temp	10	38.077	1.836	-9.939	0.293	48.016	1.82
Backhousia myrtifolia	Hot	Temp	10	44.747	1.56	-10.079	0.525	54.826	1.668
Backhousia myrtifolia	Ref	Temp	10	38.159	1.994	-9.515	0.295	47.674	2.176
Banksia integrifolia	Cold	Temp	10	43.504	1.732	-10.318	0.493	53.822	1.863
Banksia integrifolia	Combo	Temp	10	43.157	1.248	-9.886	0.333	53.043	1.116
Banksia integrifolia	Hot	Temp	10	46.297	0.684	-9.988	0.359	56.285	0.782
Banksia integrifolia	Ref	Temp	10	42.744	0.65	-10.416	0.554	53.077	0.804
Carex appressa	Cold	Temp	10	49.837	0.519	-14.891	0.992	63.239	1.488
Carex appressa	Combo	Temp	10	51.629	0.967	-14.409	1.2	66.174	1.662
Carex appressa	Hot	Temp	10	51.457	0.684	-14.339	1.255	65.796	1.335
Carex appressa	Ref	Temp	10	49.76	0.473	-15.308	0.764	65.068	0.888
Lomandra longifolia	Cold	Temp	10	51.04	0.465	-11.7	0.299	61.57	1.225
Lomandra longifolia	Combo	Temp	10	51.702	0.439	-13.263	0.674	64.965	0.522
Lomandra longifolia	Hot	Temp	10	50.256	0.598	-12.439	0.848	62.696	1.254
Lomandra longifolia	Ref	Temp	10	49.674	0.611	-13.27	0.461	63.096	0.519
Melaleuca hypericifolia	Cold	Temp	10	43.458	0.648	-11.307	0.588	54.765	0.799
Melaleuca hypericifolia	Combo	Temp	10	45.984	0.636	-11.625	0.851	57.609	1.258
Melaleuca hypericifolia	Hot	Temp	10	47.988	0.534	-9.586	0.755	57.574	1.133
Melaleuca hypericifolia	Ref	Temp	10	45.197	0.592	-11.209	0.63	56.406	0.874
Pittosporum undulatum	Cold	Temp	10	44.847	1.418	-10.204	0.296	55.051	1.436
Pittosporum undulatum	Combo	Temp	10	48.542	0.379	-9.935	0.384	58.477	0.644
Pittosporum undulatum	Hot	Temp	10	48.409	0.593	-9.775	0.539	58.185	0.691
Pittosporum undulatum	Ref	Temp	10	45.264	0.664	-10.394	0.475	55.788	0.609

Acacia aneura	Cold	Des	10	49.097	0.889	-11.705	0.513	60.802	1.288
Acacia aneura	Combo	Des	10	49.587	0.486	-11.73	0.455	61.317	0.824
Acacia aneura	Hot	Des	10	50.444	0.432	-11.736	0.409	62.18	0.684
Acacia aneura	Ref	Des	8	48.612	0.885	-11.159	0.873	59.684	1.103
Acacia salicina	Cold	Des	10	46.562	1.227	-11.201	0.369	57.763	1.021
Acacia salicina	Combo	Des	10	49.526	0.591	-12.716	0.602	62.242	0.632
Acacia salicina	Hot	Des	10	49.790	0.329	-10.44	0.577	60.23	0.502
Acacia salicina	Ref	Des	10	46.894	0.776	-12.39	0.407	59.284	0.639
Acacia victoriae	Cold	Des	10	48.432	1.28	-12.419	0.5	60.851	1.465
Acacia victoriae	Combo	Des	10	48.835	0.913	-12.968	0.66	61.803	1.028
Acacia victoriae	Hot	Des	10	49.904	0.568	-11.493	0.427	61.397	0.718
Acacia victoriae	Ref	Des	6	48.13	1.219	-11.386	0.297	59.52	1.007
Capparis mitchelli	Cold	Des	10	47.134	0.495	-12.234	0.604	59.369	0.909
Capparis mitchelli	Combo	Des	10	49.58	0.536	-13.271	0.665	62.851	0.946
Capparis mitchelli	Hot	Des	10	49.075	0.471	-11.768	0.677	60.843	0.835
Capparis mitchelli	Ref	Des	10	47.349	0.51	-11.929	0.35	59.278	0.573
Casuarina pauper	Cold	Des	10	47.416	0.169	-11.339	0.79	58.755	0.759
Casuarina pauper	Combo	Des	10	48.15	0.777	-12.902	0.532	61.052	0.903
Casuarina pauper	Hot	Des	10	49.771	0.431	-11.01	0.487	60.781	0.74
Casuarina pauper	Ref	Des	10	46.553	0.737	-10.56	0.775	57.113	1.146
Dodonaea viscosa	Cold	Des	10	43.925	0.852	-11.192	0.722	55.117	1.136
Dodonaea viscosa	Combo	Des	10	45.823	0.686	-9.503	0.974	55.326	1.196
Dodonaea viscosa	Hot	Des	10	44.447	0.973	-11.137	0.557	55.584	1.068
Dodonaea viscosa	Ref	Des	10	39.897	1.001	-10.148	0.471	48.97	1.528
Eucalyptus largiflorens	Cold	Des	10	46.59	0.786	-10.729	0.767	57.319	0.975
Eucalyptus largiflorens	Combo	Des	10	46.14	0.562	-10.737	0.796	56.877	0.767
Eucalyptus largiflorens	Hot	Des	10	49.405	0.734	-10.406	0.984	59.811	0.986
Eucalyptus largiflorens	Ref	Des	10	43.699	0.827	-9.492	0.797	53.191	1.084
Flindersia maculosa	Cold	Des	10	41.143	1.239	-11.971	0.543	52.826	1.101
Flindersia maculosa	Combo	Des	10	43.032	1.572	-12.124	0.631	55.271	1.649
Flindersia maculosa	Hot	Des	10	43.617	1.251	-11.971	0.869	55.769	1.215
Flindersia maculosa	Ref	Des	10	39.352	0.929	-11.258	0.539	50.86	1.126
Eucalyptus pauciflora	Cold	Alp	10	42.649	1.055	-6.801	0.157	49.45	1.005
Eucalyptus pauciflora	Combo	Alp	10	44.437	1.109	-7.582	0.25	52.019	1.045
Eucalyptus pauciflora	Hot	Alp	10	48.502	0.584	-7.231	0.131	55.733	0.656
Eucalyptus pauciflora	Ref	Alp	10	42.673	1.71	-6.781	0.371	49.395	1.779
Ewartia nubigena	Cold	Alp	6	47.959	0.554	-9.9912	0.722	57.951	1.001

Ewartia nubigena	Combo	Alp	8	48.383	0.308	-15.005	0.894	63.388	1.029
Ewartia nubigena	Hot	Alp	6	48.314	0.733	-12.27	1.188	60.584	1.869
Ewartia nubigena	Ref	Alp	8	47.199	0.411	-16.265	0.687	63.464	0.547
Leptorhynchos squamatus	Cold	Alp	10	49.54	0.256	-10.045	1.16	59.585	1.092
Leptorhynchos squamatus	Combo	Alp	10	48.763	0.263	-13.472	1.204	62.236	1.333
Leptorhynchos squamatus	Hot	Alp	10	48.772	0.702	-14.027	1.444	62.799	1.646
Leptorhynchos squamatus	Ref	Alp	10	47.204	0.706	-15.39	1.445	62.421	1.61
Oxylobium ellipticum	Cold	Alp	10	46.451	0.355	-9.244	0.623	55.695	0.639
Oxylobium ellipticum	Combo	Alp	10	46.722	0.522	-11.087	0.77	56.701	1.601
Oxylobium ellipticum	Hot	Alp	10	49.089	0.556	-8.708	0.537	56.926	1.237
Oxylobium ellipticum	Ref	Alp	10	47.071	0.403	-10.12	0.489	56.18	1.117
Poa costiniana	Cold	Alp	10	47.595	1.068	-15.539	0.814	63.134	1.629
Poa costiniana	Combo	Alp	10	49.193	0.57	-14.845	1.065	63.644	1.398
Poa costiniana	Hot	Alp	10	49.978	1.591	-18.431	0.447	68.409	1.581
Poa costiniana	Ref	Alp	10	47.412	0.941	-16.074	1.251	63.486	1.847
Poa heimata	Cold	Alp	10	47.699	0.735	-16.589	1.394	64.288	2.012
Poa heimata	Combo	Alp	10	48.813	0.797	-16.965	1.35	64.082	1.892
Poa heimata	Hot	Alp	10	49.19	0.306	-17.17	1.589	68.214	1.269
Poa heimata	Ref	Alp	10	48.391	0.944	-15.682	1.76	64.073	1.79
Ranunculus graniticola	Cold	Alp	10	45.808	0.633	-12.398	1.012	57.762	1.129
Ranunculus graniticola	Combo	Alp	10	46.818	0.303	-10.865	0.655	57.683	0.819
Ranunculus graniticola	Hot	Alp	10	47.181	0.401	-9.606	0.503	56.787	0.599
Ranunculus graniticola	Ref	Alp	10	44.265	0.335	-14.17	0.755	58.435	0.911
Xerochrysum subundulatum	Cold	Alp	9	47.573	1.089	-10.223	0.896	57.796	1.234
Xerochrysum subundulatum	Combo	Alp	10	47.565	0.577	-11.98	0.627	59.545	0.916
Xerochrysum subundulatum	Hot	Alp	10	48.086	0.609	-9.602	0.545	57.688	0.645
Xerochrysum subundulatum	Ref	Alp	10	47.414	0.38	-11.327	0.61	58.741	0.795

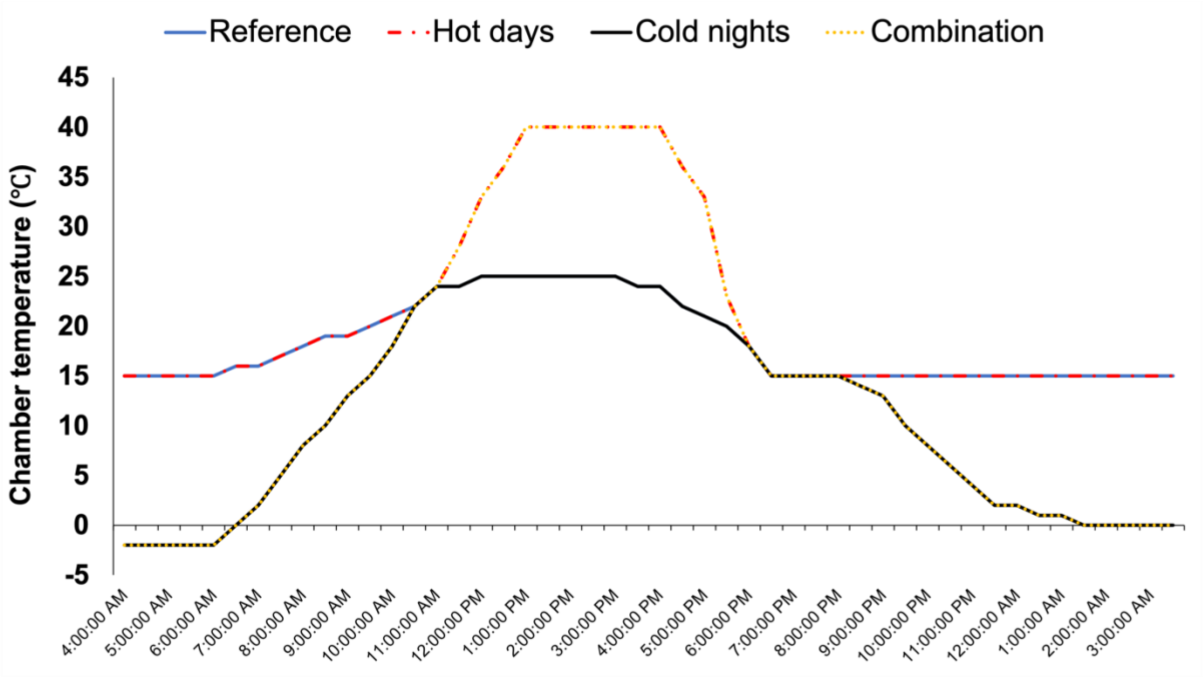

Supplementary Figure 1. Time course of the target air temperatures for each treatment across a 24hr period. Reference treatment was 25°C/15°C, hot days were 40°C/15°C, cold nights were 25°C/-2°C and the combination treatment was 40°C/-2°C.

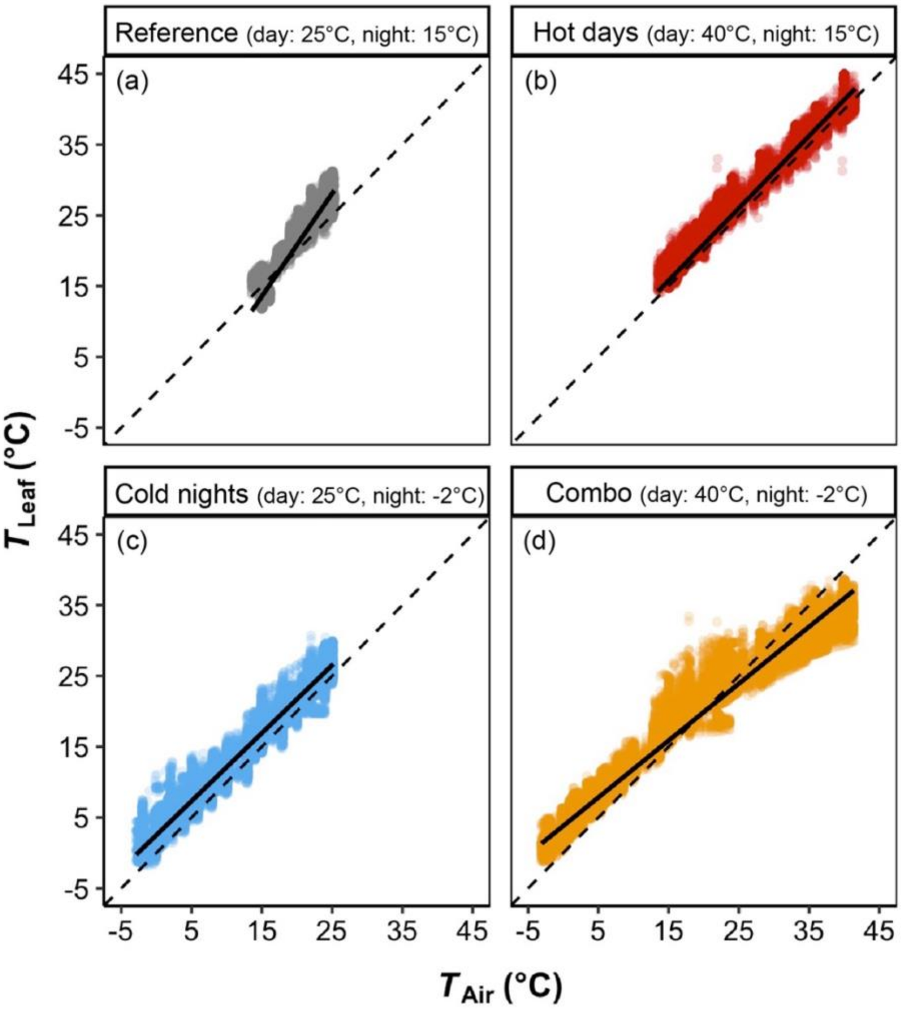

Supplementary Figure 2. The offset between the treatment chambers air temperature and leaf temperature pooled over 5 days of the extreme events for 15 of the 24 species in the study. If points fall above the dashed 1:1 line, leaf temperature exceeded air temperature, and if points fall below the line, then leaf temperature was cooler than air temperature. Linear models fit to each treatment pooled across all species indicated that leaves in benign conditions had slightly (but significantly) cooler night temperatures and warmer day time temperatures than air temperature, whereas plants exposed to -2°C nights were on average 2°C warmer than the chamber air temperature. All treatments had slightly warmer leaf temperatures during the day except for the combination treatment that had somewhat cooler leaves than the air temperature during the 40°C heatwave. We judged that these modest differences were not sufficient to undermine the efficacy of the treatments.

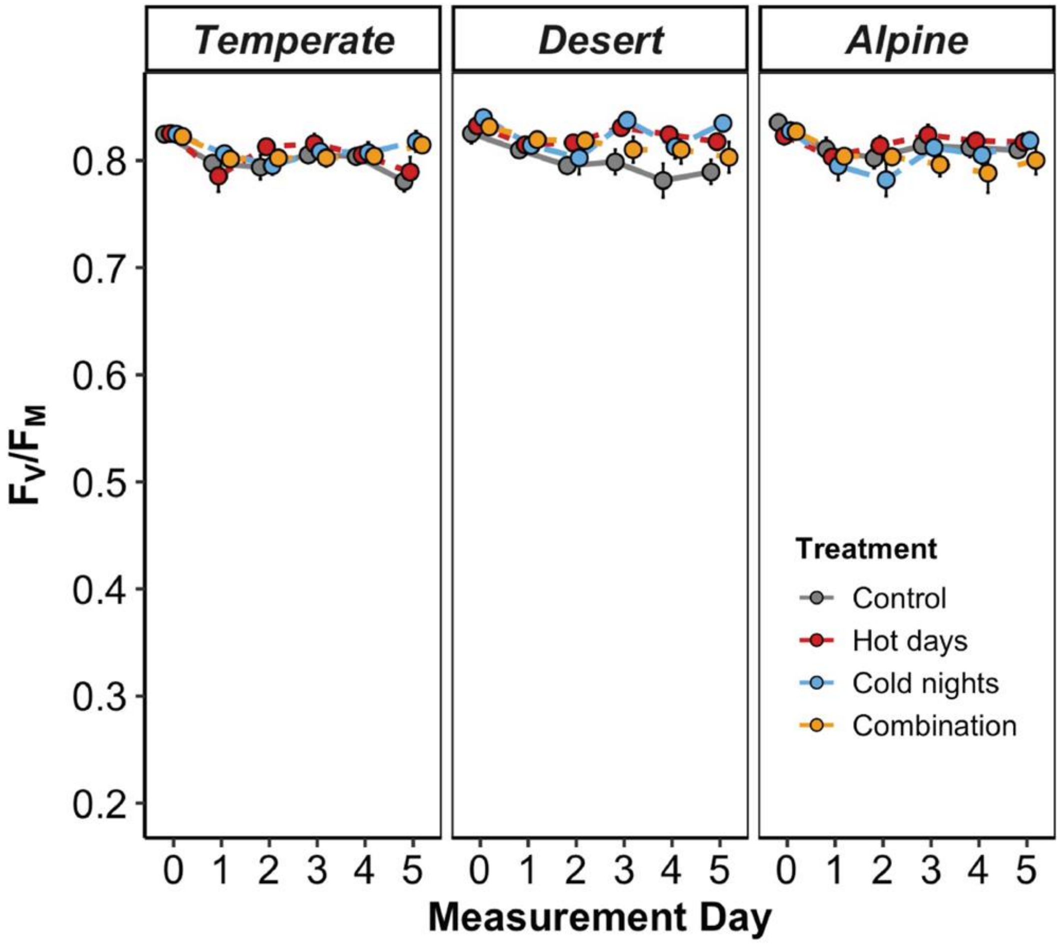

Supplementary Figure 3. Measurements of maximum quantum yield ($F_{v/}F_m$) across each day of the experiment for each treatment. No change in $F_{v/}F_m$ throughout the entirety of the experiment, highlighting plants were not damaged during extreme events but still showed strong acclimation potential when using the temperature-dependent fluorescence metric.

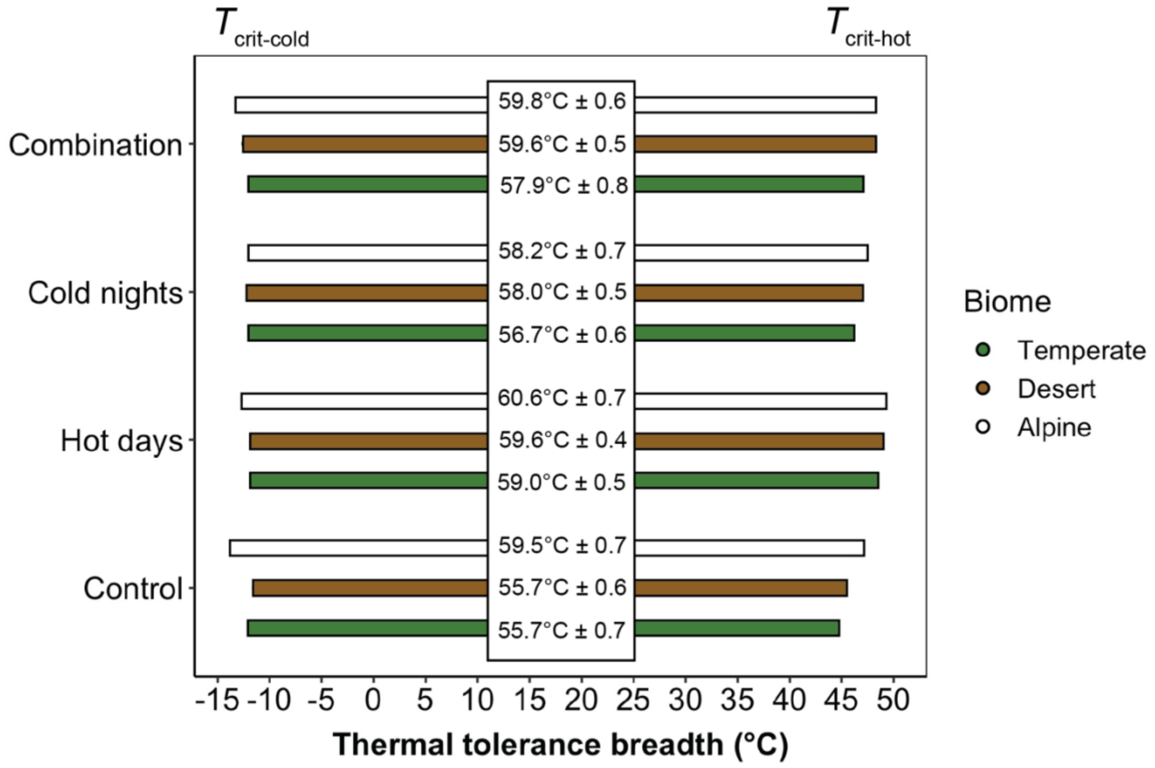

Supplementary Figure 4. Thermal tolerance breadth (TTB) for each biome and treatment plotted as the means of $T_{\text{crit-cold}}$ (left side) and $T_{\text{crit-hot}}$ (right side). Values in the box are TTB means (difference between $T_{\text{crit-hot}}$ and $T_{\text{crit-cold}}$) ± SE. All biomes had very wide TTBs with alpine plants having the widest breadth in the reference group, although these were the least responsive to the treatments. Temperate and desert plants increased TTB by almost 5°C in response to hot days for example.
